# Parâmetros da Hemodinâmica Central como Novos Biomarcadores de Risco Cardiovascular

**DOI:** 10.36660/abc.20201218

**Published:** 2020-12-01

**Authors:** José Fernando Vilela-Martin

**Affiliations:** 1 Faculdade de Medicina FAMERP São José do Rio PretoSP Brasil Faculdade de Medicina de São José do Rio Preto (FAMERP),São José do Rio Preto, SP — Brasil; 2 Clínica de Hipertensão FAMERP São José do Rio PretoSP Brasil Clínica de Hipertensão da FAMERP, São José do Rio Preto, SP — Brasil

**Keywords:** Hipertensão, Pressão Sanguínea, Hemodinâmica, Fatores de Risco, Análise da Onda de Pulso, Doenças Vasculares/prevenção e controle

Historicamente, a importância da onda de pulso arterial já era observada pelos egípcios e os chineses, antes de Cristo. O conhecimento da hemodinâmica periférica mostrou um grande avanço após a introdução da medida não invasiva da pressão arterial (PA) com o esfigmomanômetro há cerca de 120 anos e, até hoje, a PA braquial representa um excelente preditor de morbidade e mortalidade cardiovascular.^1^ No entanto, modificações da macro e da microcirculação não podem ser completamente observadas somente pela medida periférica da PA. Assim, alterações vasculares estruturais e funcionais podem ser melhor avaliadas por parâmetros da hemodinâmica central, representados por PA central, *augmentation index* e velocidade de onda de pulso (VOP),^2 , 3^ sendo a VOP o padrão-ouro na avaliação da rigidez arterial.^4^ O referencial do valor prognóstico da hemodinâmica central foi demonstrado clinicamente pelo estudo CAFE (Conduit Artery Function Evaluation Study), qual evidenciou que uma maior redução da PA central comparada à PA periférica resultou em maior redução de eventos cardiovasculares.^5^

Por sua vez, outros estudos associaram o papel da VOP à presença de lesões cardiovasculares e cerebrovasculares, de tal forma que este tema foi incluído nas diretrizes europeias de hipertensão em 2007.^6^A VOP foi usada pela primeira vez como índice clínico da elasticidade arterial em 1922, mas sua determinação demorou a ser aplicada à prática clínica porque seu registro e cálculo eram difíceis de se obter. A rigidez aórtica, medida pela VOP carótida-femoral, tem sido a mais utilizada em estudos epidemiológicos. A obtenção da VOP no segmento carotídeo-femoral é simples, não invasivo, reprodutível, amplamente aceito e clinicamente relevante, pois inclui a aorta, um segmento importante em relação aos efeitos fisiopatológicos da rigidez arterial. Atualmente, VOP pode ser considerada um biomarcador de risco cardiovascular,^7^ além de ser preditor de eventos cardiovasculares e de mortalidade.^8 , 9^

O conceito de marcadores de lesões orgânicas foi introduzido nas últimas décadas. Biomarcador é uma medida variável que se apresenta como substância encontrada em amostra biológica ou pode ser avaliada por exames de imagem. Um biomarcador pode refletir fisiopatologia da doença, predizer futuros eventos ou indicar a presença da doença subclínica ou clínica. Um biomarcador também pode ser medido para avaliar a resposta ao tratamento instituído. Ocasionalmente, um marcador pode funcionar como um fator etiológico ou de risco.^10^

Reconhecidamente, a PA é um biomarcador universal para hipertensão arterial sistêmica (HAS). A medida da PA define a condição de hipertensão, orienta a abordagem terapêutica e avalia respostas ao tratamento instituído. Biomarcadores adicionais oferecem a possibilidade de reclassificar indivíduos, especialmente nas categorias de risco intermediário, com maior ou menor risco de lesão de órgão-alvo do que o estimado somente pela PA. Assim, fornecer informações independentes da PA e de outros fatores de risco clássicos é um dos requisitos básicos para um biomarcador que pode servir como um instrumento de reestratificação de risco, fato proposto no artigo apresentado nesta edição. Fagundes e colaboradores investigaram a relação entre biomarcadores de lesões subclínicas, tendo por base a relação entre VOP e biomarcadores de hipertrofia ventricular esquerda (espessura do septo interventricular e da parede posterior do ventrículo esquerdo, e diâmetro do átrio esquerdo) e um marcador vascular [espessura íntima média carotídea (EIM)]. Eles demonstraram que a VOP se correlacionou com a EIM e com os parâmetros ecocardiográficos acima, mostrando uma associação independente com EIM, isto é, EIM acima de 1 mm aumentou em aproximadamente 4 vezes a chance de VOP maior do que 10m/s, ponto de corte acima do qual o risco de eventos cardiovasculares aumenta.^11^

Adicionalmente, o uso de outros parâmetros de hemodinâmica central, tal como a PA central, é capaz de detectar diferentes fenótipos de HAS em conjunto com a PA braquial e classificar o risco cardiovascular de forma mais fidedigna. Chuang et al.^12^ mostraram em uma população adulta quatro fenótipos distintos de PA, baseando-se nas medidas da pressão periférica e PA central, ou seja, normotensão concordante braquial e central, hipertensão isolada braquial, hipertensão isolada central e hipertensão concordante braquial e central. Também demonstraram que a elevação concordante das duas pressões levou a maior risco de doença arterial coronariana em 10 anos em comparação à elevação de somente uma das pressões avaliadas. O estudo também evidenciou que a detecção de HAS somente pelo método convencional subestimou a real prevalência de hipertensão, em comparação ao uso combinado das duas formas de avaliação da PA.^12^ Em outro estudo realizado em idosos ≥65 anos, a hipertensão combinada braquial e central esteve significantemente associada com lesões cardíacas (hipertrofia ventricular esquerda e disfunção diastólica), vasculares (VOP) e renais (relação albumina/creatinina) em comparação às medidas isoladas, tanto periférica quanto central.^13^

Assim, a PA periférica continua sendo o melhor biomarcador no manuseio de pacientes com HAS; todavia, fornece informações incompletas sobre a patogênese e o comprometimento de órgãos-alvo, e pode não representar o melhor meio de avaliação da resposta terapêutica instituída. Novas modalidades de biomarcadores, representados pelos parâmetros da hemodinâmica central, ajudarão a individualizar estratégias preventivas e terapêuticas em indivíduos com hipertensão. A PA não será substituída por outros biomarcadores, mas pode ser complementada por marcadores que forneçam informações adicionais.^14^
