# Supplementary material for: Parâmetros da Hemodinâmica Central como Novos Biomarcadores de Risco Cardiovascular
Source: Arq Bras Cardiol. 2020 Dec 1;115(6):1133–4. [Article in Portuguese] doi: 10.36660/abc.20201218 (PMC8133714; doi:10.36660/abc.20201218)
Supplement: Translation

# Translation: Parameters of Central Hemodynamics as New Biomarkers of Cardiovascular Risk

**Authors:** José Fernando Vilela-Martin

**Affiliations:** 1 Brazil Faculdade de Medicina de São José do Rio Preto (FAMERP), São José do Rio Preto, SP — Brazil; 2 Brazil Clínica de Hipertensão da FAMERP, São José do Rio Preto, SP — Brazil

**Keywords:** Hypertension, Blood Pressure, Hemodynamic, Risk Factors, Pulse Wave Analysis, Vascular Diseases/prevention and control

Historically, the importance of the arterial pulse wave was already observed by the Egyptians and the Chinese, before Christ. The knowledge of peripheral hemodynamics showed great progress after the introduction of non-invasive blood pressure (BP) measurement using the sphygmomanometer about 120 years ago and, to date, brachial BP represents an excellent predictor of cardiovascular morbidity and mortality.^1^However, changes in macro and microcirculation cannot be completely observed only by peripheral BP measurement. Thus, structural and functional vascular changes can be better assessed by central hemodynamic parameters, represented by central BP, augmentation index and pulse wave velocity (PWV),^2 , 3^with OPV being the gold standard in the assessment of arterial stiffness.^4^The reference prognostic value of central hemodynamics was clinically demonstrated by the CAFE study (Conduit Artery Function Evaluation Study), which showed that a greater reduction in central BP compared to peripheral BP resulted in a greater reduction in cardiovascular events.^5^

In turn, other studies associated the role of PWV with the presence of cardiovascular and cerebrovascular lesions, so this topic was included in the European guidelines for hypertension in 2007.^6^PWV was first used as a clinical index of arterial elasticity in 1922, but its determination too long to be applied to clinical practice because its registration and calculation were difficult to obtain. Aortic stiffness, measured by carotid-femoral PWV, has been the most used in epidemiological studies. Obtaining PWV in the carotid-femoral segment is simple, non-invasive, reproducible, widely accepted and clinically relevant, as it includes the aorta, an important segment in relation to the pathophysiological effects of arterial stiffness. Currently, PWV can be considered a biomarker of cardiovascular risk^7^and is a predictor of cardiovascular events and mortality.^8 , 9^

The concept of organic lesion markers has been introduced in the past decades. A biomarker is a variable measure that presents itself as a substance found in a biological sample or can be evaluated by imaging tests. A biomarker can reflect the pathophysiology of the disease, predict future events or indicate the presence of subclinical or clinical disease. A biomarker can also be measured to assess the response to the treatment established. Occasionally, a marker can function as an etiological or risk factor.^10^

BP is recognized as a universal biomarker for systemic arterial hypertension (SAH). BP measurement defines the condition of hypertension, guides the therapeutic approach and assesses responses to the treatment established. Additional biomarkers offer the possibility of reclassifying individuals, especially in the intermediate risk categories, with a greater or lesser risk of target organ damage than that estimated by BP alone. Thus, providing information regardless of BP and other classic risk factors is one of the basic requirements for a biomarker that can serve as an instrument for restructuring risk, as proposed in the article presented in this edition. Fagundes et al. investigated the relationship between biomarkers of subclinical lesions based on the relationship between PWV and biomarkers of left ventricular hypertrophy (interventricular septum thickness and left ventricular posterior wall thickness, and left atrial diameter) and a vascular marker [carotid intima-media thickness (cIMT)]. They demonstrated that PWV correlated with cIMT and with the echocardiographic parameters above, showing an independent association with cIMT, that is, cIMT above 1 mm increased by about 4 times the chance of PWV greater than 10 m/s, a cutoff point above which the risk of cardiovascular events increases.^11^

Besides, the use of other parameters of central hemodynamics, such as central BP, is able to detect different SAH phenotypes with brachial BP and to classify cardiovascular risk more reliably. Chuang et al.,^12^showed, in an adult population, four distinct BP phenotypes based on the measurements of peripheral pressure and central BP, that is, concordant brachial and central normotension, isolated brachial hypertension, isolated central hypertension and concordant brachial and central hypertension. They also demonstrated that the concordant increase of the two pressures led to a greater risk of coronary artery disease in 10 years compared to the increase of only one of the evaluated pressures. The study also showed that the detection of SAH by the conventional method alone underestimated the real prevalence of hypertension, compared to the combined use of the two forms of BP assessment.^12^In another study with elderly aged 65, combined brachial and central hypertension was significantly associated with cardiac (left ventricular hypertrophy and diastolic dysfunction), vascular (PWV) and renal (albumin/creatinine ratio) compared to isolated peripheral and central measures.^13^

Thus, peripheral BP remains the best biomarker in the management of patients with SAH; however, it provides incomplete information about the pathogenesis and involvement of target organs, and may not represent the best means of assessing the therapeutic response established. New modalities of biomarkers, represented by the parameters of central hemodynamics, will help to individualize preventive and therapeutic strategies in individuals with hypertension. BP will not be replaced by other biomarkers, but it can be supplemented by markers that provide additional information.^14^
